# Upregulation of miR-150* and miR-630 Induces Apoptosis in Pancreatic Cancer Cells by Targeting IGF-1R

**DOI:** 10.1371/journal.pone.0061015

**Published:** 2013-05-10

**Authors:** Lulu Farhana, Marcia I. Dawson, Farhan Murshed, Jayanta K. Das, Arun K. Rishi, Joseph A. Fontana

**Affiliations:** 1 Department of Oncology, Wayne Sate University, Detroit, Michigan, United States of America; 2 John D. Dingell VA Medical Center, Detroit, Michigan, United States of America; 3 Wayne State University, Detroit, Michigan, United States of America; 4 Karmanos Cancer Institute, Detroit, Michigan, United States of America; 5 Sanford-Burnham Medical Research Institute, La Jolla, California, United States of America; Deutsches Krebsforschungszentrum, Germany

## Abstract

MicroRNAs have been implicated in many critical cellular processes including apoptosis. We have previously found that apoptosis in pancreatic cancer cells was induced by adamantyl retinoid-related (ARR) molecule 3-Cl-AHPC. Here we report that 3-Cl-AHPC-dependent apoptosis involves regulating a number of microRNAs including miR-150* and miR-630. 3-Cl-AHPC stimulated miR-150* expression and caused decreased expression of c-Myb and IGF-1R in the pancreatic cancer cells. 3-Cl-AHPC-mediated reduction of c-Myb resulted in diminished binding of c-Myb with IGF-1R and Bcl-2 promoters, thereby causing repression of their transcription and protein expression. Over-expression of miR-150* also resulted in diminished levels of c-Myb and Bcl-2 proteins. Furthermore, the addition of the miRNA inhibitor 2′-O-methylated miR-150 blocked 3-Cl-AHPC-mediated increase in miR-150* levels and abrogated loss of c-Myb protein. Knockdown of c-Myb in PANC-1 cells resulted in enhanced apoptosis both in the presence or absence of 3-Cl-AHPC confirming the anti-apoptotic property of c-Myb. Overexpression of miR-630 also induced apoptosis in the pancreatic cancer cells and inhibited target protein IGF-1R mRNA and protein expression. Together these results implicate key roles for miR-150* and miR-630 and their targeting of IGF-1R to promote apoptosis in pancreatic cancer cells.

## Introduction

Following their initial discovery in 1993, microRNAs have been studied for their purpose by a large number of authors [1]. The ability of microRNAs to regulate the expression of a wide variety of genes at the post-transcriptional level has been well documented [2]. These small RNA molecules are conserved and expressed in a large number of organisms including *Homo sapiens* and play important roles in the regulation of crucial biological processes including cell proliferation, differentiation and apoptosis.

MicroRNAs are transcribed in the nucleus primarily by RNA polymerase as long primary transcripts (pre-microRNAs). These molecules are then processed in the nucleus by RNAse III Drosha into 70- to 100-nucleotides pre-microRNAs and then exported into the cytoplasm where they are further processed by the RNAse III Dicer to generate double stranded RNAs (dsRNA) of approximately 22 nucleotides [3]. Whether, there is degradation of the antisense strand at this point is controversial. Recent evidence strongly suggests that the reverse mRNA strand may not be degraded and may play a significant role in the regulation of a number of cellular functions [4]. The remaining mature single-stranded micro RNA inhibits translation by joining a complex that binds complementary to the 3′-UTR of the target gene. Through complimentary binding, specific microRNAs have been shown to target a number of genes enhancing or inhibiting their expression, resulting in pleiotropic effects on a number of cellular functions [5].

Dysregulation of microRNA expression has been associated with cancer initiation and progression by regulating expression of tumor suppressors and oncogenes. It has been previously demonstrated that the microRNA profiles found in pancreatic carcinoma tissues differ significantly from those found in normal pancreatic tissue or in pancreatitis [6]. It has been hypothesized that enhanced or diminished expression of specific microRNAs may be powerful approach in the therapy of a number of malignancies [5]. A number of approaches to modulate microRNA expression have been devised. The adamantly-substituted retinoid related (ARR) molecules have been found to induce apoptosis in a variety of malignant cells both *in vitro* and *in vivo* [7]. A number of mechanisms have been proposed through which apoptosis is achieved by this class of molecules [7].

We have previously demonstrated that ARRs are potent inducers of apoptosis in pancreatic cancer cells – a dismal disease with an often devastating prognosis [8]. We hypothesized that the exposure of cells to the 3-Cl-AHPC may result in the modulation of microRNA expression and that this differential microRNA expression may contribute to ARR-mediated inhibition of cellular proliferation and cell death induction. In the present series of experiments, we demonstrate that the ARRs indeed modulate microRNAs expression and that this enhancement of specific microRNA expression miR-150* and miR-630 results in the down regulation of IGF-1R protein which are important mediator of cellular growth and apoptosis induction.

## Materials and Methods

### Reagents and Cells

(*E*)-4-[3-(1-adamantyl)-4-hydroxyphenyl]-3-chlorocinnamic acid (3-Cl-AHPC) was synthesized as previously described [9]. Human pancreatic carcinoma cell lines, PANC-1 and MiaPaCa-2 were obtained from the American Type Culture Collection (ATCC, Rockville, MD) and maintained in DMEM-F12 medium containing 10% FBS and 100 µg/ml gentamycin. DMEM-F12 medium, fetal bovine serum (FBS) and lipofectamine 2000 were purchased from Invitrogen (Carlsbad, CA). Antibodies and their sources were as follows: antibodies for flow cytometry, CD44-PE, CD24-FITC from BD Biosciences (San Jose, CA); anti- IGF-1Rβ, anti-SMAD1, anti-cleaved caspase 3 (Asp-175), anti-caspase 3 were from Cell Signaling Technology (Boston, MA); anti-EGFR, anti-CDK6, anti-c-Myb and anti-Bcl2 antibodies (Santa Cruz Biotechnology, Santa Cruz, CA); Cdc14A and ABCC1 from Abcam, Cambridge, MA and Thermo Scientific, Rockford, IL, respectively; and anti-α-tubulin and β-actin antibody (Oncogene Research Products, Boston, MA). Puromycin was purchased from Sigma-Aldrich (St. Louise, MO).

### miRNA microarray analysis and validation

PANC-1 cells were exposed to 1 µM 3-Cl-AHPC for 30 h. Total RNA from 3-Cl-AHPC treated and untreated cells was extracted with TRIzol reagent (Invitrogen, Carlsbad, CA) and then sample preparation, hybridization to G4470A 15K Human miRNA Microarray (V2) (Agilent Technologies) and analysis were performed by Asuragen, Inc. (Austin, TX). For miRNA validation, total RNA was purified with RNeasy Mini kit (Qiagen) and 100 ng of RNA was used to prepare cDNA using TaqMan microRNA reverse transcription kit (Applied Biosystems) using specific microRNA primers. The miRNA sequence specific primer and probe of miR-134, miR-150*, miR-630, miR-345, miR-382, miR410, and miR129-5P, endogenous control RNU6B and TaqMan 2× PCR Master mix for TaqMan microRNA assays were purchased from Applied Biosystems and followed the protocol as described by manufacturer's instruction. Real-time qRT-PCR and analysis was performed in Applied Biosystems 7500 Real Time PCR system. Data analysis was performed with ΔCt values of miRNAs from each sample were calculated by normalizing with internal control RNU6B and each value were mean of three replicates.

### mRNA Quantitation

Total RNA was prepared from PANC-1 cell lines using TRIzol as recommended by the manufacturer and purified using the Rneasy Mini Kit (Qiagen). For real time PCR, cDNA was prepared with the SuperScript III First-Strand cDNA synthesis system for RT-PCR (Invitrogen) and analyzed in triplicate using the 2× SYBR Green PCR Master Mix (Applied Biosystems) and the ABI Prism 7500 sequence detection system. PCR consisted of 40 cycles of 95°C for 10 min and then 95°C for 15 sec, 60°C for 60 sec. The oligonucleotide primer sets were synthesized from Integrated DNA technology Inc. (Coralville, IA). The primer set for each gene is listed below:

EGFR, forward 5′- CTTTCGATACCCAGGACCAAG-3′; reverse 5′- CAACTTCCCAAAATGTGCCC -3′; CDK6, forward 5′- TGCACAGTGTCACGAACAGA -3′;

reverse 5′- ACCTCGGAGAAGCTGAAACA-3′; ABCC1,

forward 5′- AGGTGGACCTGTTTCGTGAC-3′;

reverse 5′- ACCCTGTGGATCCACCAGAAG-3′; SMAD1 forward

5′- CAACAATCGTGTGGGTGAAG -3′; reverse 5′- TCCGGTTAACATTGGAGAGC -3′;

CDC14A, forward 5′- GCACACCCAGTGACAACATC -3′; reverse

5′- CCTTCACTGGATGGTCGATT -3′; IGF-1R, forward

5′- AACCCCAAGACTGAGGTGTG -3′; reverse 5′- TGACATCTCTCCGCTTCCTT -3′;

c-Myb, forward 5′- GTCCGAAACGTTGGTCTGTT -3′; reverse

5′- GGCAGTAGCTTTGCGATTTC-3′; Bcl2, forward 5′- ATGTGTGTGGAGAGCGTCAA -3′

reverse 5′- ACAGTTCCACAAAGGCATCC -3′; β-actin, forward

5′-TCCTTCCTGGGCATGGAG-3′; reverse 5′-AGGAGGGGCAATGATCTT-3′.

### miRNAexpression vector construction and shRNA-c-Myb plasmid

For construction of miR-150* expression vector, pre-miR-150* sequences were synthesized from Integrated Technologies Inc. and annealed miR-150* sequence was clones into pcDNA6.2-GW/EmGFP-miR vector from BLOCK-iT-Poll II miR RNAi expression Vector kit (Invitrogen). miR-150* sequence in the plasmid backbone pre-miR-150* was confirmed by sequencing. miR-150* expression vector stably transfected into PANC-1 cells using lipofectamine 2000 and the stable cell lines were selected with puromycin.Vector containing scrambled sequence was used as a control.

Small hairpin (sh)-RNA Myb expression vector was constructed by directionally cloning 5′-*Bam*H I and 3 *EcoR* I overhang nucleotides in a pSIREN-RetroQ vector according to the manufacturer's instructions (Clontech, Mountain View, CA). The gene silencing target sequences were from the coding sequence of the PubMed Accession numbers NM_001130172 and sh-RNA sequences, 5′- TGAAGAAGCTGGTGGAACA -3′ (Myb-KD1) and 5′- ACAGATGACTGGAAAGTTA -3′ (Myb-KD2),5′- CAGAAGAGGAAGACAGAAT-3′ (Myb-KD3) were synthesized from Integrated DNA technology Inc. shRNA regions in the plasmid backbone were confirmed by sequencing. shRNA-Myb knockdown plasmids were stably transfected into PANC-1 cells using lipofectamine 2000 and the stable cell lines were selected with puromycin. The sh-vector- containing scrambled sequences in pSIREN-RetroQ vector was used as a control. For miR-630 overexpression, pEP-has-miR-630 expression vector was purchased from Cell Biolabs. Inc. (San Diego, CA). To inhibit miR-150* and miR-630 miRNAs, cells were transfected with antisense O'methylated miR-150* (OME-miR-150) and miR-630 (OME-miR-630) sequences; the sequences was synthesized from Integrated DNA Technology Inc.

### Apoptosis and Growth inhibition

Pancreatic cancer cell lines were treated with 1 µM 3-Cl-AHPC for various indicated time. Apoptosis in cells were analyzed by flow cytometry using Annexin V-FITC binding together with propidium iodide (PI) staining (Annexin V-FITC apoptosis Detection Kit 1, BD Biosciences, San Diego, CA). Data acquisition was done on a FACS Calibur flow cytometer (BD) and analyzed with CellQuest software (BD Biosciences). Induction of cell death of miR-630 transiently transfected PANC-1 cells was assessed by Cell Death Dectection ELISAplus kit (Roche Applied Science, Indianapolis, IN). Inhibition of cell growth were determined by 3-(4,5-dimethylthiazol-2yl)-2,5-diphenyltetrazolium bromide (MTT) assay. The cells were seeded on 96-well plates at a density of 5×10^4^ cells/well in a volume of 200 µl culture medium and pre-miR-630 expression vector, miR vectors were transfected with lipofectamine. After 48 h of transfection, 25 µl/well of MTT (5 mg/ml) were added to the medium and incubated for 4 h and MTT precipitates were solubilized with 200 µl DMSO and the plates read on a BioTeK Synergy HT (BioTeK Instrument Inc., Vermont) at an absorbance 590 nm. All experiments were performed in quadruplicate to determine means and standard deviations.

### Western blots

Cells were extracted with lysis buffer containing 25 mM Tris-Cl buffer (pH 8.0), 150 mM NaCl, 0.2% nonidet P-40, 10% glycerol 10 mM NaF, 8 mM β-glycerophosphate, 0.2 mM Na_3_VO_4_, 1 mM DTT, and 10 µl/ml protease inhibitor cocktail (Sigma Aldrich, St. Louise, MO) and Western blots were performed as we previously described [10].

### Chromatin Immunoprecipitation (ChIP) assay

PANC-1 control cells were treated with 1 µM 3-Cl-AHPC for 24 h and the formaldehyde cross-linking and immunoprecipitation analysis was performed using a described method [10]. Chromatin lysate was incubated with 1 µg of anti-c-Myb antibody overnight at 4°C and immunoprecipitate complexes were collected with Dynal magntic beads (invitrogen). The following primers were used in in the ChIP assay samples for IGF-1R and Bcl2 promoter region,

IGF-1R, forward, 5′- AGGGGAATTTCATCCCAAAT-3′; reverse,

5′-AGGAAAAGTTCCCGCAGTG - 3′; Bcl2, forward,

5′- CAGAGGAGGGCTTTCTTTCTTCTT-3′; reverse, 5′-CCCGGCCTCTTACTTCATTCT -3′

Real-Time PCR was carried out as described previously in mRNA quantitation section. The data analysis was performed with ΔCt values from each c-Myb bound control. 3-Cl-AHPC treated samples were calculated by normalizing with input control and input treated value and each value was the mean of three replicates. The PCR products were run by electrophoresis on a 2% agarose gel and visualized by ethidium bromide staining and image captured by Gel Logic 2200 Imaging system, Molecular Imaging System Carestream Health, Inc.

### Fluorescence-activated cell sorting (FACS) of CD44^+^/CD24^+^ cells and sphere formation

Cells were grown to 70–80% confluence, trypsinised and washed with sorting buffer (1×PBS, 5% FCS). The cells were resuspended with 100 µl sorting buffer and incubated with 15–20 µl anti-CD44-PE and anti-CD24-FITC primary antibodies for 30 min at ice. The cells were washed and resuspended in 500 µl of sorting buffer and sorted using flow cytometry FACSAria system (BD Immunocytochemistry Systems, Franklin lakes, NJ).

Sorted CD44^+^/CD24^+^ cells were suspended in serum-free stem cell medium containing DMEM/F12 (1∶1) supplemented with B27 (Life Technologies, Gaithersburg, MD), 20 ng/ml EGF (Biomol International, Plymouth, PA), 20 ng/ml fibroblast growth factor (Biomol International, Plymouth, PA), and 100 µg/ml gentamycin. Approximately 150–200 cells per well were seeded in an ultra low-attachment 96-well plate (Corning Inc, Lowell, MA). 3-Cl-AHPC (1.0 µM) was added the day after cells were plated or after 7 days of sphere formation. Spheres were photographed and measured utilizing an Olympus microscope (OLYMPUS CKX41) and Olympus microscope digital camera with DP2-BSW software (Olympus soft imaging solutions GmbH, Germany). All statistics were performed using VassarStats web statistical software (Richard Lowry, Poughkeepsie, NY, USA). One-way analysis of variance (ANOVA) was performed to detect any differences between groups of sphere control, 3-Cl AHPC treated spheres. If the result of the ANOVA was significant (**P<0.01 vs control), pair wise comparisons between the groups were made by a post-hoc test (Tukey's HSD procedure). The significance level was set at **P<0.01 vs control and *P<0.05 vs control. Square brackets were used in the figures to indicate treatments that are significantly different from the control.

## Results

### 3-Cl-AHPC mediated modulation of specific microRNAs

**Figure 1:**
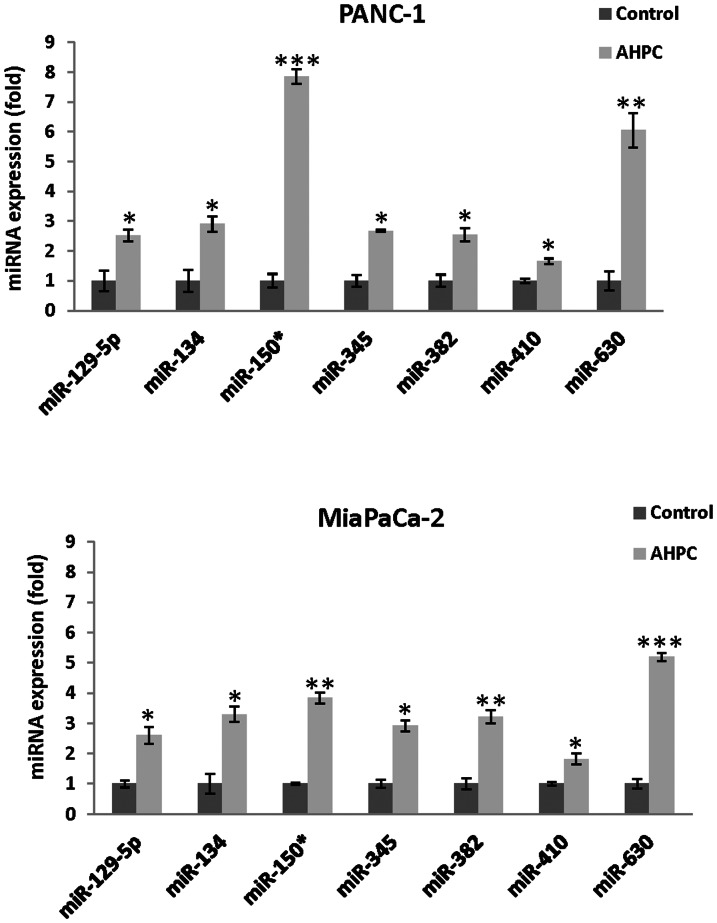
3-Cl-AHPC mediated modulation of microRNAs in pancreatic cancer cells. MicroRNAs were validated by quantitative Real time PCR. Cells were treated with 3-Cl-AHPC for 24 h. Detail methodologies were as described in Materials and Methods. The error bars represent the mean of three separate determinations ± the standard deviation (SD). *, ** and *** (<0.05, <0.01 and <0.001) were significantly different in comparison between control and treated samples using *t*-Test.

PANC-1 cell exposure to 1 µM 3-Cl-AHPC modulated expression of a number of microRNAs (Table 1 microarray analysis). Up-regulation of miR-134, miR-150*, miR-630, miR-345, miR-382, miR-410 and miR-129-5p and down-regulation of miR-202 and miR-578 were also observed (not shown). Further evidence of 3-Cl-AHPC-mediated miRNA modulation was obtained by real-time-PCR using a Taqman probe. Real-time PCR demonstrated a statistically significant increase in the expression of the various microRNA species at 24 h (Figure 1) in

**Table 1:** 3-Cl-AHPC mediated modulation of microRNAs and target genes in PANC-1 cells.

miRNA	Fold change (Up-regulated)	Predicted miRNA Target genes (TargetScanHuman 5.1)
miR-134	3.96	EGFR
miR-150*	3.91	Myb
miR-630	4.78	IGF-1R, Cdc14A
miR-345	3.8	SMAD1
miR-382	5.5	MRP1 (ABCC1)
miR-410	2.33	CPEB3
miR-129-5P	2.1	CDK6

Legends: Epidermal growth factor receptor (EGFR), insulin-like growth factor 1 receptor (IGF-1R).

Multidrug resistance-associated protein 1 (MRP1),

cytoplasmic polyadenylation element binding protein 3 (CPEB3),

cyclin-dependent kinase 6 (CDK6).

**Figure 2:**
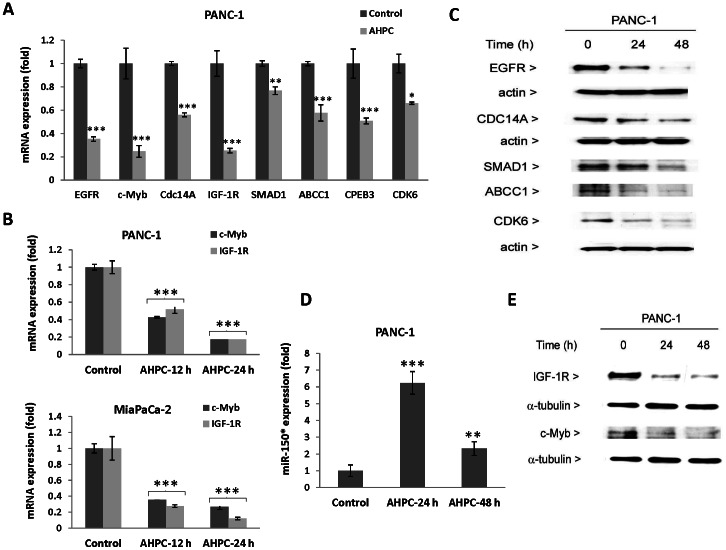
3-Cl-AHPC mediated expression of microRNA targets in pancreatic cancer cells. (A and B) Reduced expression of mRNA by SYBR-green RT-PCR; (C and E) decreased proteins levels were demonstrated by Western blots analysis. D). 3-Cl-AHPC exposure increased miR-150* expression and subsequently decreased expression of c-Myb and IGF-1R proteins (E). The error bars represent the mean of three separate determinations ± the standard deviation (SD). *, ** and *** (<0.05, <0.01 and <0.001) were significantly different in comparison between control and treated samples using *t*-Test.

PANC-1 and MiaPaCa-2 cells. We also determined whether 3-Cl-AHPC modulation of microRNA expression resulted in up- or down-regulation of the genes targeted by these microRNAs; target genes were identified using the TargeScanHuman 5.0 data base. Modulation of the targeted genes including EGFR, c-Myb, CDC14A, IGF-1R, SMAD1, ABCC1, CPEB3, and CDK6 at both the mRNA (Figure 2A and B) and the protein level (Figure 2C and E) was demonstrated within 24 h of 3-Cl-AHPC exposure. We also found that exposure of the PANC-1 cells to 3-Cl-AHPC for 24 h or 48 h increased expression of miR-150* while decreasing expression of c-Myb protein and IGF-1R in cells (Figure 2D and E).

### Over-expression of miR150* enhanced apoptosis by regulating c-Myb and IGF-1R and inhibited CD44^+^/CD24^+^ stem cell-like spheres formation

**Figure 3:**
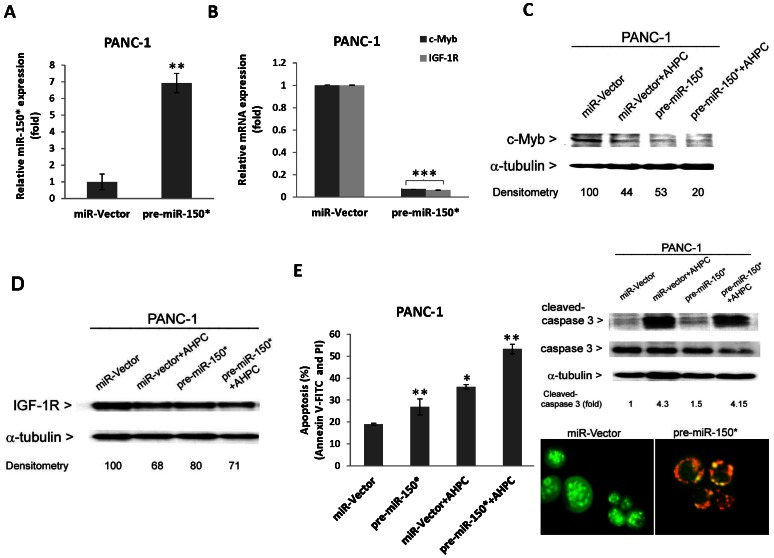
Overexpression of pre-miR-150* reduced target proteins and induced apoptosis in pancreatic cancer cells. (A) Increased expression of pre-miR-150* expression vector and pre-miR-150* reduced expression of c-Myb and IGF-1R mRNA and protein in pre-miR-150* stably transfected cells (B–D). Cells were treated with 1 µM 3-Cl-AHPC for 24 h. (E) pre-miR-150* induced apoptosis by itself and enhanced 3-Cl-AHPC mediated apoptosis in PANC-1 cells. Cells were transiently transfected with pre-miR-150* expression vector for 24 h and then exposed to 3-Cl-AHPC for 24 h. Induction of apoptosis was assessed using Annexin V-FITC labeling with propidium iodide (PI) staining in cells and the error bars represent the mean of three separate determinations ± the standard deviation (SD). All treated samples are significantly different from miR-vector. * (<0.05), ** (<0.01 ) and *** (<0.001 ) significantly different in comparison to miR-vector by *t*-Test. Over-expression of miR-150* induced activation of caspase 3 as indicated by cleaved caspase 3 fragments in pre-miR-150* transiently transfected cells (upper right panel). pre-miR-150* expressed PANC-1 cells induced apoptosis as indicated in acridine orange/ethidium bromide stained cells in lower right panel. Cells were transfected with pre- miR-150* for 48 h and then stained and photographed using Olympus fluorescence microscope digital camera software and DP2-BSW software.

**Figure 4:**
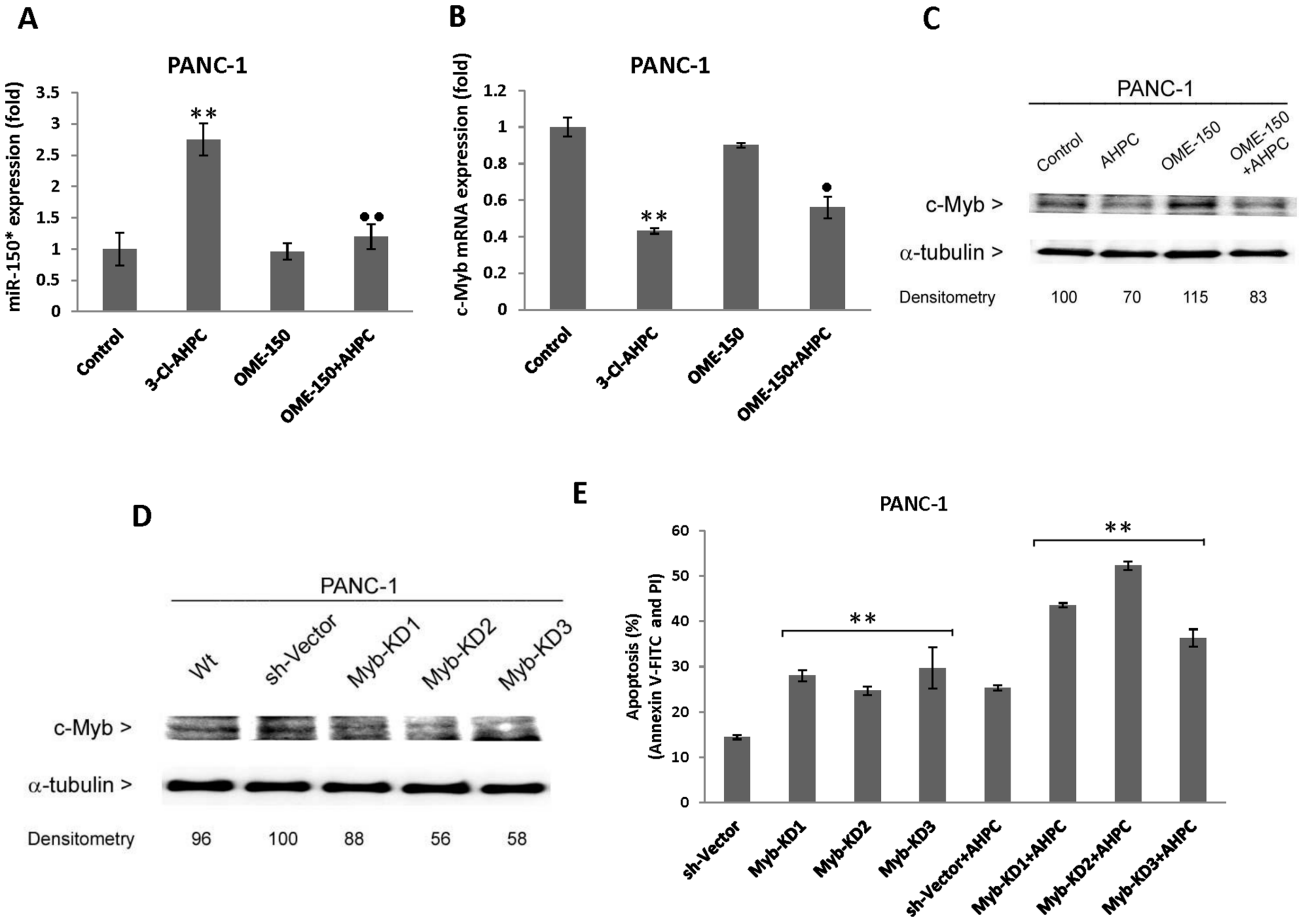
Inhibitor of miR-150* blocked 3-Cl-AHPC-mediated decreased of c-Myb in PANC-1 cells. (A) Inhibitor 2′-O-methylated miR-150* (OME-150) inhibited 3-Cl-AHPC mediated miR-150* expression in transiently transfected cells. (B and C) miR-150 inhibitor blocked the 3-Cl-AHPC mediated c-Myb degradation. Cells were treated with 1 µM 3-Cl-AHPC for 24 h. (D) Knock down of c-Myb expression in cells. (E) Knock down of c-Myb enhanced the apoptosis in cells. Cells stably transfected with three sh-Myb knockdown expression vectors and scrumble sequence control vector were exposed to 3-Cl-AHPC for 24 h. Induction of apoptosis was assessed using Annexin V-FITC labeling with propidium iodide (PI) staining in cells and the error bars represent the mean of three separate determinations ± the standard deviation (SD). All treated samples and sh-Myb are significantly different from control and sh-vector; • and ••, ** (<0.05 and <0.01 respectively) are significantly different by *t*-Test and • represented the comparison between 3-Cl-AHPC to OME-150*+3-Cl-AHPC treated samples.

Previous research has shown that miR-150 targets proto-oncogene transcription factor c-Myb during multiple steps of lymphocyte development [11]. In order to further demonstrate miR-150* modulation of c-Myb and IGF-1R in the PANC-1 cells, pre-miR-150* was stably over-expressed using a pre-miR-150* expression vector (pre-miR-150*) in PANC-1 cells resulting in a 7-fold increase in pre-miR-150* expression (Figure 3A); pre-miR-150* over-expression resulted in a 80% inhibition of IGF-1R and c-Myb mRNA levels, a 50% decreased expression of c-Myb protein levels and a 32% decrease in IGF-1R expression (Figure 3B–D). In addition, both increased expression of pre-miR-150* by itself and in the presence of 3-Cl-AHPC significantly enhanced apoptosis in the PANC-1 cells (Figure 3E). Pre-miR-150* activated caspase 3 1.5-fold, showing proteolytic cleaved-caspase 3 fragments (Figure 3E, upper right panel) and inducing the DNA fragmentation (Figure 3E, lower right panel). These results together implicate a pro-apoptotic role of miR-150 through its regulation of its target genes. Incubation of the PANC-1 cells with the inhibitor 2′-O-meyhylated miR-150* antisense (OME-miR150*) blocked 3-Cl-AHPC-mediated elevation of miR-150* levels and decreased in c-Myb expression strongly. This result suggests that 3-Cl-AHPC exerts its effects on c-Myb and IGF-1R expression through modulation of microRNAs (Figure 4A–C).

We hypothesized that c-Myb was anti-apoptotic in the PANC-1 cells and knockdown of c-Myb would enhance apoptosis of the cells both in the absence and the presence of 3-Cl-AHPC. Three clones of sh-Myb knockdown were generated in PANC-1 cells and low levels of c-Myb expression were found (Figure 4D). Knockdown of Myb demonstrated enhanced apoptosis in sh-Myb-KD cells. The addition of 3-Cl-AHPC to these cells also resulted in significantly greater levels of apoptosis than noted with cells transfected with only the vector (Figure 4E).

**Figure 5:**
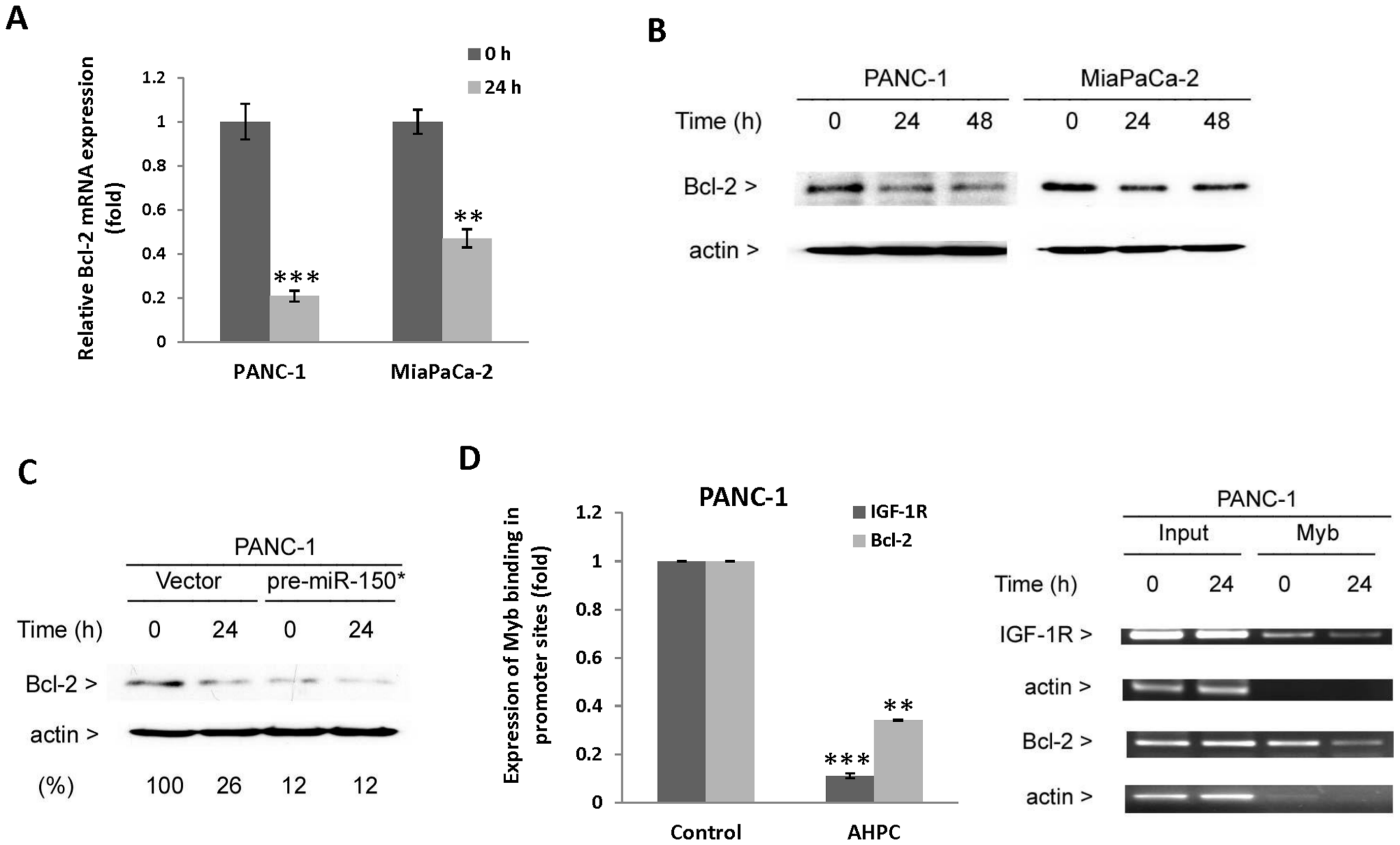
3-Cl-AHPC mediated decreased c-Myb binding to Bcl-2 and IGF-IR promoters resulted in reduced protein expression. (A and B) 3-Cl-AHPC decreased Bcl-2 mRNA and protein expression. (C) Over-expression of pre-miR-150* reduced Bcl-2 protein expression. (D) 3-Cl-AHPC decreased c-Myb- binding to IGF-1R and Bcl-2 promoter sites in chromatin immunoprecipitation assay. Cells were exposed to 3-Cl-AHPC for 24 h. The error bars represent the mean of three separate determinations ± the standard deviation (SD). ** and *** (<0.01 and <0.001) were significantly different in comparison between control to 3-Cl-AHPC treated mRNA and Myb binding in promoter sites using *t*-Test.

Bcl-2 also plays a major role in antagonizing apoptosis by a number of agents [12]. It has been previously reported that c-Myb has a binding site in the Bcl-2 promoter and is a positive regulator of Bcl-2 expression. Decreased c-Myb levels in the inducible dominant-negative Myb protein FDCP-mix clone A4 cells resulted in decreased Bcl-2 expression [13]. We therefore examined Bcl-2 expression in cells exposed to 3-Cl-AHPC. We found significantly decreased Bcl-2 mRNA and protein expression in both PANC-1 and MiaPaCa-2 cells (Figure 5A and B). In addition, over expression of the pre-miR-150* resulted in decreased Bcl-2 protein expression suggesting it's regulated by miR-150* through c-Myb (Figure 5C). Chromatin immunoprecipitation assays were preformed to elucidate the mechanism by which 3-Cl-AHPC decreases Bcl-2 and IGF-1R expression. 3-Cl-AHPC exposure resulted in a significant decrease in the binding of c-Myb to the IGF-1R and Bcl-2 promoters (Figure 5D).

**Figure 6:**
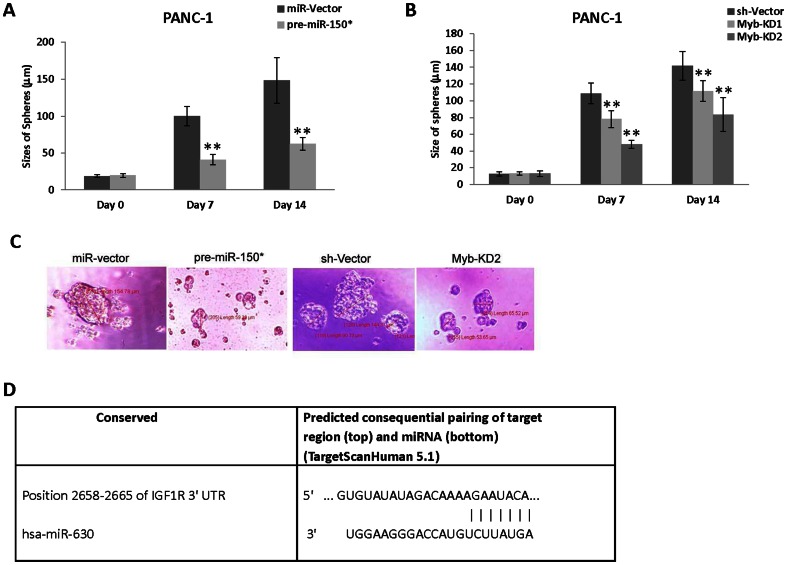
Expression of pre-miR-150* and knockdown of c-Myb inhibited CD44^+^/CD24^+^ stem-cell like spheres formation in cells. (A and C) Pre-miR-150* inhibited sphere formation in CD44^+^/CD24^+^ cells. (B and C) c-Myb knockdown inhibited sphere formation in CD44^+^/CD24^+^ PANC-1 cells. For sphere formation, the CD44^+^/CD24^+^ cells were sorted by flow cytometry from stably transfected pre-miR150* and sh-Myb knock down cells, respectively. The sizes of spheres were photographed and measured on a 100 µm scale and magnification 400× using Olympus fluorescence microscope digital camera software and DP2-BSW software. The error bars represent the mean of 15 sphere determinations ± the standard deviation. ** was significantly different in comparison to control spheres. Data were analyzed by ANOVA; Tukey HSD test for multiple comparisons. **P<0.0001 vs control. (D) Scheme showing predicted target conserved site of miR-630 in the 3′UTR of IGF-1R.

We have previously found that PANC-1 cells enriched for CD24/CD44 positivity possessed the stem cell-like feature of forming spheres in non-adherent growth conditions [8]. The addition of 3-Cl-AHPC to these spheres inhibited their growth and resulted in the induction of apoptosis. We therefore assessed the effect of elevated levels of pre-miR-150* and knockdown of c-Myb expression on the growth of these spheres (Figures 6A, B and C). Both elevated levels of pre-miR-150* and knockdown of c-Myb expression resulted in a significant growth inhibition of spheres at 7 and 14 days. Similarly, Zhang *et al.* [14] have recently shown that increased miR-150 expression inhibits CD133 positive liver cancer stem cells through its inhibition of c-Myb expression.

### Target protein IGF-1R is regulated by miR-630

**Figure 7:**
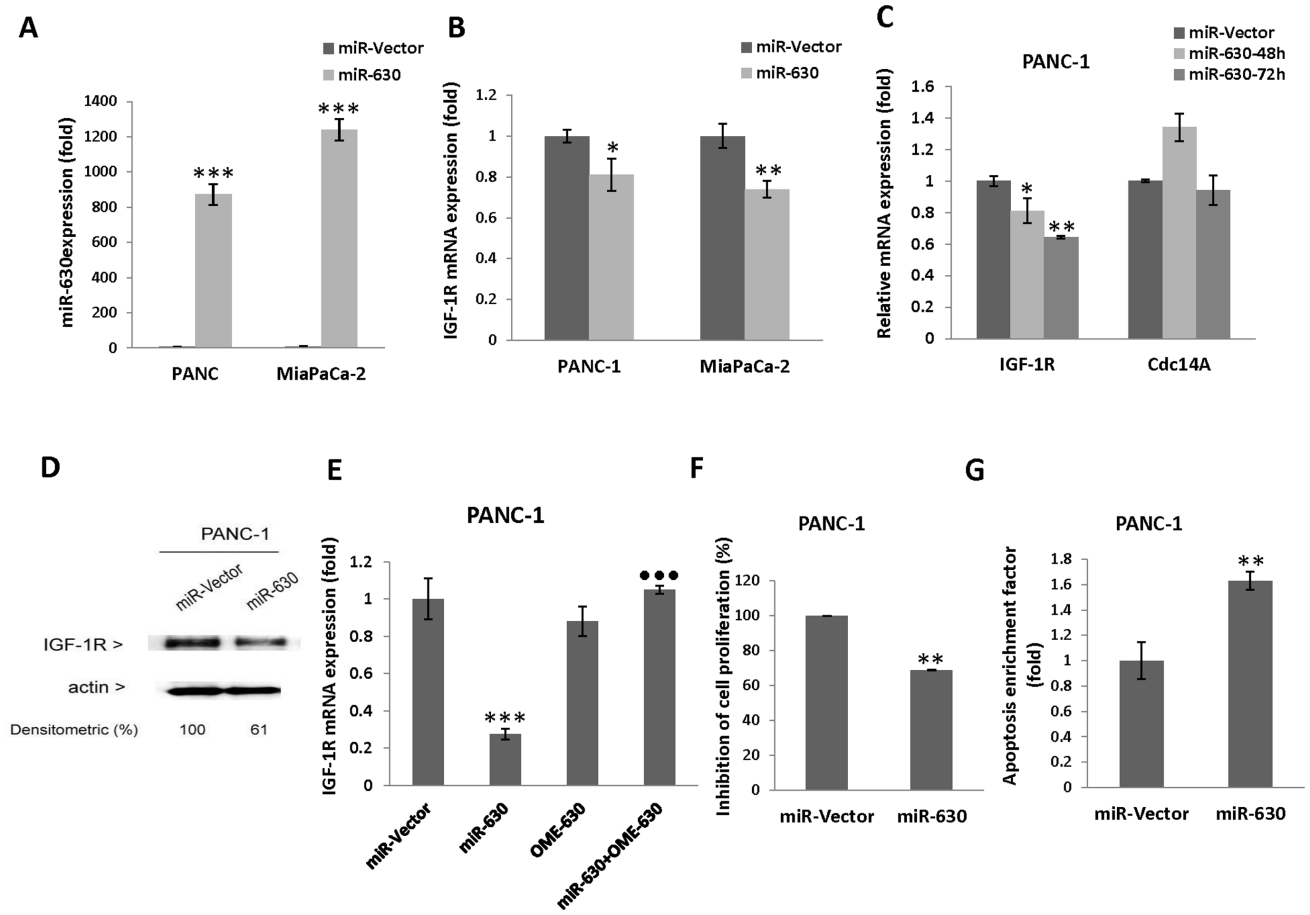
Over-expression of pre-miR-630 reduced IGF-1R expression and inhibited cell growth and induced apoptosis in cells. (A) pre-miR-630 expression in PANC-1 and MiaPaCa-2 cells. (B and C) Increased expression of pre-miR-630 decreased IGF-1R mRNA but not Cdc14A in cells. (D). Decreased IGF-1R protein expression in pre-miR-630 expressed cells. (E) Inhibitor 2′-O-methylated miR-630 (OME-630) blocked the IGF-1R mRNA degradation. Cells were transiently transfected with pre-miR-630 for 48 h (F and G) pre-miR-630 expression inhibited growth and induced apoptosis in PANC-1 cells. Inhibition of cell proliferations was evaluated after 48 h of pre-miR-630 transiently transfected cells by MTT assay and expressed as percent of that inhibited at a time relative to the miR-vector control. The error bars represent the mean of four separate determinations ± the standard deviation (SD). *, ** and *** (<0.05, <0.01 and <0.001) were significantly different in comparison between miR-vector and miR-630; • was highly significant and represented the comparison between miR-630 to miR-630+OME-630 using *t*-Test. Apoptosis of miR-630 cells was evaluated after 48 h of transfected cells. The cell death was measured by cytoplasmic histone-associated-DNA-fragments by ELISA (enrichment factor = OD of miR-630 expressed lysate/OD of miR-vector, OD at 405 nm-490 nm).

Galluzzi *et al.* have shown that miR-630 regulates cisplatin induced growth arrest by modulating cell cycle inhibitor p27^Kip1^ and induces apoptosis in non-small cell lung cancer [14]. We have found that PANC- 1 cells exposed to 3-Cl-AHPC enhanced miR-630 expression 6-fold. Using TargetScanHuman 5.1 software (Table 1), we found potential miR-630 target genes IGF-1R and Cdc14A. miR-630 pairs to a 7 nucleotide conserved region located in position 2658–2665 of IGF-1R 3′-UTR (Figure 6D). Over-expression of pre-miR-630 reduced IGF-1R mRNA and protein expression in transiently transfected cells (Figure 7A–D) whereas there was no change in the mRNA level target gene Cdc14A. 3-Cl-AHPC decreased the Cdc14A mRNA and protein expression (Figure 2A and C). The mechanism by which 3-Cl-AHPC triggered decreased Cdc14A expression remains to be determined. The antisense inhibitor 2′-O-methylated miR-630 blocked pre-miR-630 mediated IGF-1R mRNA degradation indicated that a miR-630 target gene is IGF-1R (Figure 7E). In addition, over-expression of pre- miR-630 enhanced inhibition and apoptosis significantly in PANC-1 cell (Figures 7F and G). These results demonstrate the important role of miR-630 in the induction of apoptosis in pancreatic cancer cells.

## Discussion

The importance of microRNA expression in maintaining normal cellular physiology has been well documented [15]. Inhibition of microRNA processing has been shown to enhance cellular transformation [16]. The incomplete knockdown of Dicer 1 in mouse lung adenocarcinoma cells resulted in increased cellular proliferation and invasiveness as well as enhanced *in vivo* tumor formation [16]. Furthermore, decreased microRNA expression in mouse embryonic fibroblasts resulted in incomplete transformation of the cells [16]. The mechanisms by which microRNA expression decreases in the malignant cells as opposed to the normal cells is not clear. Recently, epigenetic regulation of microRNA expression has been demonstrated in a variety of tumors [17]. MicroRNA genes were silenced in human tumors by aberrant hypermethylation of CpG islands adjacent to and surrounding the microRNA genes and by acetylation of the microRNA gene associated histones [17]. It has been found that a higher proportion of known microRNA genes have been found to be epigenetically regulated through methylation (11.6%) than that found in the protein-encoding genes (1–2%) [18], [19].

In the present publication, we have demonstrated that 3-Cl-AHPC modulates expression of a number of microRNAs that regulate the translation and degradation of mRNA. These mRNA encode the expression of proteins previously demonstrated to have important physiological roles in cellular proliferation and death. We have found that 3-Cl-AHPC exposure resulted in the up-regulation of a number of microRNAs including miR-150* and miR-630. The significance of modulating of these microRNAs was demonstrated by observing subsequent modulation of their predicted target genes- including EGFR, c-Myb, SMAD1, ABCC1, CPEB3, IGF-1R and CDK6.

3-Cl-AHPC up-regulation of miR-150* resulted in the decreased expression of c-Myb and IGF-1R. Both proteins play critical roles in enhancing cellular proliferation. Over-expression of IGF-1R has been found in a variety of malignancies including pancreatic cancer [20]. Inhibition of IGF-1R utilizing small molecule kinase inhibitors has resulted in reduced pancreatic cancer growth [21]. We have previously shown that knock down of IGF-1R expression reduced pancreatic cancer cell proliferation while also enhancing 3-Cl-AHPC-mediated apoptosis [8], [22]. We have recently demonstrated that the expression of the microRNA miR-223 also suppresses cell proliferation by targeting IGF-1R. Enhanced miR-223 resulted in a decrease in IGF-1R expression as well as inhibition of the downstream Akt/mTOR/p70S6K pathway.

3-Cl-AHPC-mediated 7-fold increase in miR-150* decreased IGF-1R and c-Myb expression. In order to further demonstrate miR-150* modulation of c-Myb and IGF-1R in the PANC-1 cells, we expressed pre-miR-150* in the cells and this resulted in a marked inhibition of IGF-1R and c-Myb mRNA and protein levels (Figure 3). Futhermore, addition of the miRNA inhibitor OME-150 with 3-Cl-AHPC blocked 3-CL-AHPC increase in miR-150* levels and 3-Cl-AHPC mediated decrease in c-Myb protein. These results strongly suggest that the 3-Cl-AHPC-mediated decrease c-Myb levels was due to the 3-Cl-AHPC increase in pre-miR-150* expression. Moreover, enhanced expression of miR-150* in the PANC1 cells resulted in enhanced apoptosis in the cells both in the presence and absence of 3-Cl-AHPC. Thus enhanced mir-150* expression and its down-regulation of c-Myb and IGF-1R have a significant effect on cell survival. Bcl-2 has been found to function as a negative regulator of apoptosis and has been found to play an important role in tumor cell survival [12], [23]. Bcl-2 down-regulation has been shown to be important in the induction of tumor cell death [12], [23]. We found that 3-Cl-AHPC exposure resulted in down-regulation of Bcl-2 expression by decreasing c-Myb expression and binding to the Bcl-2 promoter.

Aberrant expression of miR-150 has been reported in a number of malignant cells [24]–[28]. miR-150 was found to be down-regulated in lymphoma and leukemia while its expression has been found to be increased in gastric and colorectal carcinomas. miR-150 appears, however, to have pleiotropic effects on malignant cells. Enhanced miR-150 expression promotes gastric carcinoma cell proliferation while it functions as a tumor suppressor in malignant lymphoma [26], [28]. Recently, it has been found that miR-150 directly targets the Muc4 3′mRNA 3′-UTR [22]. Binding of miR-150 to this region resulted in a marked decrease expression of MUC4 mRNA and protein. This decreased expression was shown to have a marked inhibitory effect on the pancreatic cell growth, migration, invasion and clonogenicity [24].

EGFR plays an important role in pancreatic cancer cellular proliferation, metastatic spread, and apoptosis [29]. Elevated expression of EGFR has been found to be an independent poor prognostic factor in patients with pancreatic cancer [30]. EGFR is targeted by miR-133, decreasing expression of EGFR mRNA and protein in NSCLC cells [31]. We have found that exposure of the pancreatic cancer cells to 3-Cl-AHPC resulted in 4-fold increase in miR-134 levels with an associated marked inhibition of EGFR mRNA and protein levels. Previous research has shown that cisplatin (CDDP) induced a 5-fold increased expression of miR-630 at 12 h [14]. pre-miR-630 arrested cell growth at G0-G1 in lung cancer NSCLC A549 cells. We have found that 3-Cl-AHPC up-regulates of miR-630 expression. pre-miR-630 reduced target protein IGF-1R levels and induced growth inhibition and apoptosis in PANC-1 cells. These results suggested that IGF-1R is the target gene of miR-630.

Modulation of miRNA levels by a number of agents has been demonstrated to function as cancer chemopreventive agents [32]. Epigallocatechin gallate an important cancer chemopreventative agent of green tea has been shown to upregulate miR-16 with subsequent down-regulation of Bcl-2 in hepatocarcinoma cells [33]. Sun *et al.* have recently shown that the antitumor agent curcumin which has been shown to inhibit the proliferation and induce apoptosis of a variety of tumor types may mediate the death of pancreatic cancer cells through its upregulation of the expression of miR-22 and the subsequent targeting of the transcription factor SP1 and estrogen receptor 1 [34]. Similarly, the anti-tumor effects of the natural antioxidant revesterol and the isoflavone genistein have been partially or wholly attributed to their ability the levels of a variety of miR levels [33], [35]. More direct approaches have been utilized to modulate miRNA levels both *in vitro* and *in vivo* [36]. These approaches have included the use of anti miRNA oligonucleotides (AMO) miRNA sponges – that have multiple miRNA binding sites thus possessing the capacity to sequester multiple miRNA - and miRNA masks - that are complementary to the 3′-untranslated region of the target miRNA and result in the competitive inhibition of miRNA modulation of its target genes [36], [37] A number of microRNA delivery systems have been developed and have shown promise both in vitro and in vivo in their ability to enhance specific miRNA expression in the malignant cells resulting in the inhibition of tumor growth [38], [39].

In this report we show that 3-Cl-AHPC modulated exposure to PANC-1 cells modulate miRNAs that appear to play key roles in growth inhibition and apoptosis induction in pancreatic cancer cells. Specifically, we've shown that up-regulation of miR-150* and miR-630 is important to the inhibition of IGF-1R, which inhibits growth and induces apoptosis in pancreatic cancer cells. This study broadly supports the framework that microRNAs are essential for apoptosis induction and growth inhibition in pancreatic cancer. Further understanding microRNAs regulation may have promising clinical implications for the treatment of cancer.
